# The tissue inhibitor of metalloproteinases-1 (TIMP-1) promotes survival and migration of acute myeloid leukemia cells through CD63/PI3K/Akt/p21 signaling

**DOI:** 10.18632/oncotarget.13664

**Published:** 2016-11-26

**Authors:** Dorian Forte, Valentina Salvestrini, Giulia Corradi, Lara Rossi, Lucia Catani, Roberto M. Lemoli, Michele Cavo, Antonio Curti

**Affiliations:** ^1^ Department of Experimental, Diagnostic and Specialty Medicine (DIMES), Institute of Hematology “L. and A. Seràgnoli”, University of Bologna, Bologna, Italy; ^2^ Clinic of Hematology, Department of Internal Medicine (DiMI), University of Genoa, Genoa, Italy

**Keywords:** acute myeloid leukemia, inflammation, microenvironment, migration, TIMP-1

## Abstract

We and others have shown that the Tissue Inhibitor of Metalloproteinases-1 (TIMP-1), a member of the inflammatory network exerting pleiotropic effects in the bone marrow (BM) microenvironment, regulates the survival and proliferation of different cell types, including normal hematopoietic progenitor cells. Moreover, TIMP-1 has been shown to be involved in cancer progression. However, its role in leukemic microenvironment has not been addressed. Here, we investigated the activity of TIMP-1 on Acute Myelogenous Leukemia (AML) cell functions. First, we found that TIMP-1 levels were increased in the BM plasma of AML patients at diagnosis. *In vitro*, recombinant human (rh)TIMP-1 promoted the survival and cell cycle S-phase entry of AML cells. These kinetic effects were related to the downregulation of cyclin-dependent kinase inhibitor p21. rhTIMP-1 increases CXCL12-driven migration of leukemic cells through PI3K signaling. Interestingly, activation of CD63 receptor was required for TIMP-1's cytokine/chemokine activity. Of note, rhTIMP-1 stimulation modulated mRNA expression of Hypoxia Inducible Factor (HIF)-1α, downstream of PI3K/Akt activation. We then co-cultured AML cells with normal or leukemic mesenchymal stromal cells (MSCs) to investigate the interaction of TIMP-1 with cellular component(s) of BM microenvironment. Our results showed that the proliferation and migration of leukemic cells were greatly enhanced by rhTIMP-1 in presence of AML-MSCs as compared to normal MSCs. Thus, we demonstrated that TIMP-1 modulates leukemic blasts survival, migration and function via CD63/PI3K/Akt/p21 signaling. As a “bad actor” in a “bad soil”, we propose TIMP-1 as a potential novel therapeutic target in leukemic BM microenvironment.

## INTRODUCTION

Acute Myelogenous Leukemia (AML) is a clonal disorder which originates from a rare population of leukemia stem cells (LSCs) [1]. Over the last years major efforts have been made to improve the clinical outcome of AML patients; however, their overall prognosis is still poor especially for patients harboring unfavorable biologic factors at diagnosis [2, 3].

Recent reports have unveiled a pathogenetic link between inflammation and oncogenesis [4–7]. Whereas this finding is well-accepted for solid tumors, the relationship between inflammatory networks and leukemogenesis has not been fully elucidated. Nonetheless, recent data highlight how hematopoietic stem progenitor cells (HSPCs) fate is dictated by intrinsic and extrinsic factors and how HSPCs may actively sense danger signals and pro-inflammatory cytokines [8–10]. Moreover, the cross-talk between leukemic cells and BM stromal cells may create a suitable environment that promotes malignant transformation and disease progression. Several factors and pathways have been implicated and described [11, 12]. Among them, the tuned regulation of the balance between synthesis and degradation of extra-cellular matrix (ECM) macromolecules by matrix metalloproteinases (MMPs) and their inhibitors is critical [13]. Indeed, an altered MMPs/Tissue Inhibitor of MetalloProteinases-1 (TIMP-1) expression or activity affects steady state hematopoiesis and results in increased cell proliferation, thus favoring oncogenesis, including leukemogenesis [14]. Indeed, TIMP-1 levels increase in response to inflammation [15]. Initially described as an endogenous inhibitor of MMPs and the membrane-bound metalloproteinase ADAM-10, TIMP-1 also displays cytokine-like functions, mediated by the engagement of a specific membrane receptor, namely CD63 [16]. CD63 is known as a member of the tetraspanins, that are a superfamily of cell surface-associated membrane proteins involved in cell activation, adhesion, differentiation and tumor invasion [17]. In breast cancer and melanoma cells, CD63 is abundantly expressed and interacts with TIMP-1 at cell surface, resulting in activation of cell survival signaling [18, 19]. At the functional level, TIMP-1 exerts pleiotropic effects in the BM microenvironment, such as the regulation of cellular proliferation, apoptosis and differentiation [19–21]. In particular, we previously found that TIMP-1 regulates the function of HSPCs promoting clonogenic efficiency and survival of normal CD34^+^ cells via the activation of the CD63/PI3K/pAkt signaling pathway, suggesting that TIMP-1 may be a key player in the network of pro-inflammatory factors modulating HSPC functions [22]. Similarly, in solid tumors, TIMP-1 improves the metastatic potential of cancer cells [23]. Interestingly, upregulated TIMP-1 levels are associated with unfavourable prognosis in several tumors including breast or colorectal cancer and lung carcinoma [24–26]. In hematological malignancies, TIMP-1 promotes differentiation of lymphoma cells, whereas increased TIMP-1 serum levels are associated with advanced myeloma [27–30].

In the present study we investigated, *in vitro*, the function and molecular pathways mediated by TIMP-1 in the microenvironment of AML, providing further evidence to support the relationship between inflammation and leukemia. A better definition of the mechanisms regulating the interplay of leukemic cells within bone marrow (BM) microenvironment, may have important clinical implications for the development of novel and effective therapeutical strategies.

## RESULTS

### TIMP-1 is detectable in BM and PB of AML patients

**Figure 1:**
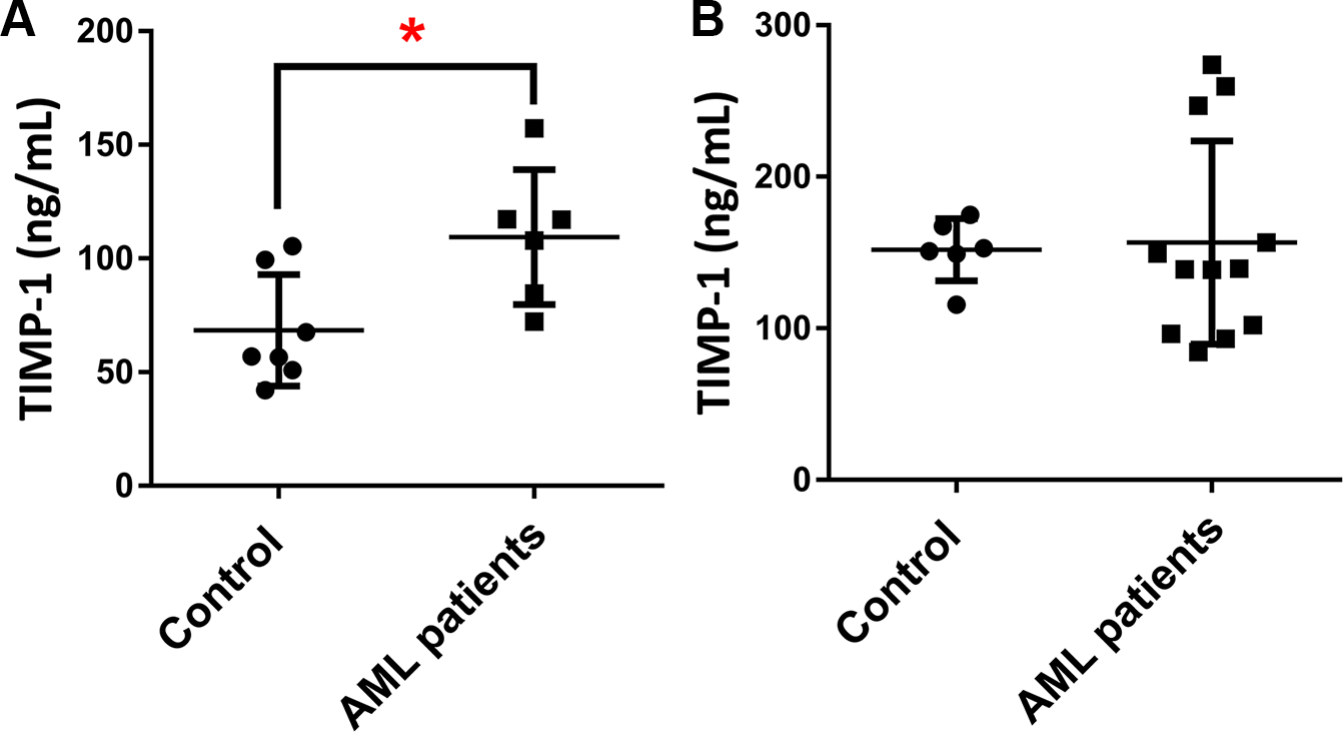
The plasma levels of TIMP-1 are increased in AML patients TIMP-1 levels were measured by ELISA in in plasma and serum of AML patients and control samples. (**A**) BM plasma concentrations of TIMP-1 were compared between AML patients at diagnosis (*n* = 6) and control samples (*p < 0.05). (**B**) In addition, PB serum concentrations of TIMP-1 were quantified in patients (*n* = 12) and compared to control samples.

In normal hematopoiesis, TIMP-1 regulates the survival and proliferation of different cell types [15, 22]. However, its role in AML BM microenvironment is not yet clear. Therefore, we first evaluated the BM plasma and PB serum level of TIMP-1 in leukemic patients at diagnosis. We found detectable amounts of TIMP-1 both in BM and PB with a median concentration of 112.6 ng/ml (range 72.25–157.3 ng/ml) and 139.4 ng/ml (range 84.47–273.9 ng/ml), respectively (Figure 1A-1B). In particular, compared with control BM samples, the plasma levels were increased in AML patients (*p ≤* 0.05). These results extend previous data in other hematological malignancies, such as pediatric ALL [32] and suggest the potential functional role of TIMP-1 within AML microenvironment. Moreover, they indicate the optimal concentration of TIMP-1 for functional studies.

### TIMP-1 increases the clonogenic efficiency of AML blasts maintaining an apoptosis-resistant phenotype

**Figure 2:**
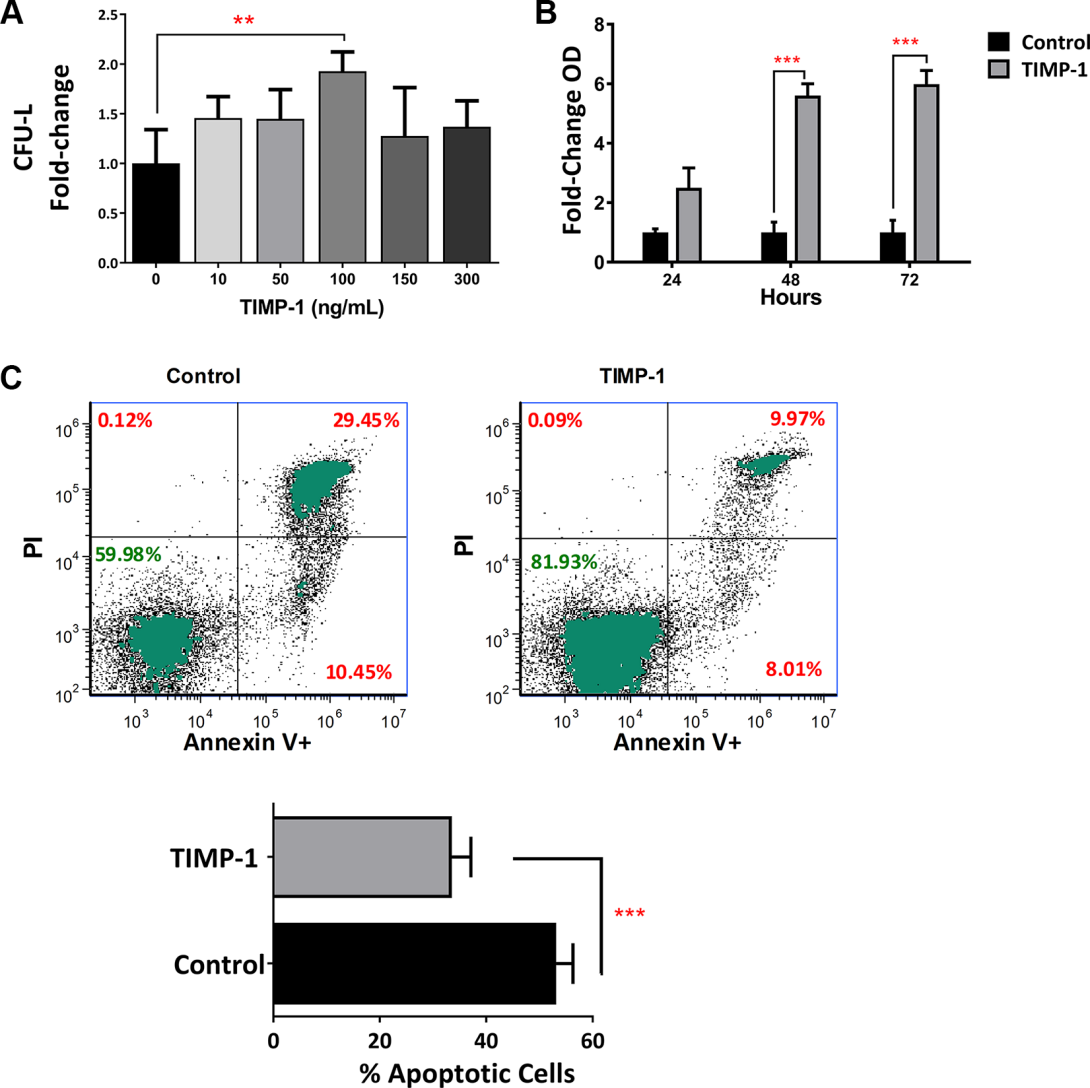
Clonogenic output and survival of leukemic blasts is positively enhanced by exposition to TIMP-1 Circulating leukemic blasts were isolated from AML patients (*n* = 12) and cultured in semisolid medium in the presence of TIMP-1. After 14 days, the total CFU-L output was assessed as above described. (**A**) The clonogenic output of the AML-derived leukemic cells was significantly stimulated by TIMP-1 (100 ng/ml, **p ≤ 0.01). No other concentration of TIMP-1 were effective. The results are expressed as growth fold change versus untreated control samples. The mean number of colonies in untreated (0 ng/ml) and treated (100 ng/ml) AML samples was 18.8 ± 5.8 vs 30.2 ± 9.2, respectively. Specifically, only 2 patients (PT #6 and #13 in the Table patients) were not responsive to TIMP-1 treatment in CFU-L assay. Data are presented as mean ± SEM of 12 patients, the error bars are the mean of each duplicate. Survival of leukemic blasts from AML patients is positively enhanced by TIMP-1. (**B**) AML blasts were cultured for 24-48-72 hrs in the presence of TIMP-1 (48–72 hrs, ***p ≤ 0.001; *n* = 3). Survival rate is evaluated by cell titer assay and expressed as fold-change taking the value of untreated cells at each time point as 1. (**C**) AML cells from 14 patients were *in vitro* treated for 2 days with TIMP-1 and the percentage of cell viability was assessed after AnnexinV/PI staining, as described in methods. Representative dot-plots showing the percentage of live, apoptotic and necrotic cells in leukemic blasts as determined by flow cytometry. Mean of percentage in apoptotic cell (*Annexin V positive and both Annexin V/ PI positive cells*) in the presence or absence of TIMP-1 (***p ≤ 0.001). The mean percentage of apoptotic cells (Annexin V positive and both Annexin V/ PI positive cells) was 53.0 ± 3.1% (range: 34.5–74.6) for control and 33.0 ± 3.5% (range: 15.4–53.0) for TIMP-1 treated cells. Data are presented as mean ± SEM.

In our previous work [22], we found that TIMP-1 increases the clonogenic efficiency of normal HSPCs isolated from umbilical CB units. Thus, we studied the effects of increasing concentration of TIMP-1 (ranging between 10–300 ng/ml) on the clonogenic output of 14 primary AML at diagnosis. As shown in Figure 2A, TIMP-1 (at 100 ng/mL) significantly increased colony formation (CFU-L) from AML patients (*p ≤* 0.01). In addition, the CFU-GM growth was positively enhanced by TIMP-1 (Supplementary Figure S1, *p ≤* 0.01).

When we tested myeloid differentiation markers (CD38 and CD11b) or hemopoietic stem/progenitor cells marker (CD34) in the blasts of AML patients, we did not find any differences between untreated or TIMP-1 treated AML samples (data not shown). We then investigated whether TIMP-1 promotes the survival of AML blasts. To this end, we treated leukemic cells with the optimal concentration of TIMP-1 (i.e. 100 ng/mL) and we evaluated cell viability at different time points by colorimetric assay (MTS assay). As summarized in Figure 2B, after 48 and 72 hours, the addition of TIMP-1 significantly increased the number of viable leukemic blasts, as evaluated as fold-change over control sample (5.6- and 5.9-fold increase, respectively; *p ≤* 0.001). Moreover, when we evaluated the apoptotic rate of leukemic blasts, we found that AML cells incubated for up to 72 hours in presence of TIMP-1 showed a significant decrease in their programmed cell death (Figure 2C). In particular, the mean percentage of apoptotic cells was reduced in presence of TIMP-1 as compared to control samples (33 ± 3.5% and 53 ± 3.14%, respectively; *p ≤* 0.001). Overall results and data from a representative example (panel C) are shown in Figure 2 and from individual patients (Supplementary Figure S2). Taken together, these data demonstrate that TIMP-1 promotes the survival and the clonogenic activity of leukemic blasts by significantly reducing apoptosis.

### TIMP-1 stimulates cell cycling of leukemic blasts in association with p21^WAF1/CIP1^ downregulation

**Figure 3:**
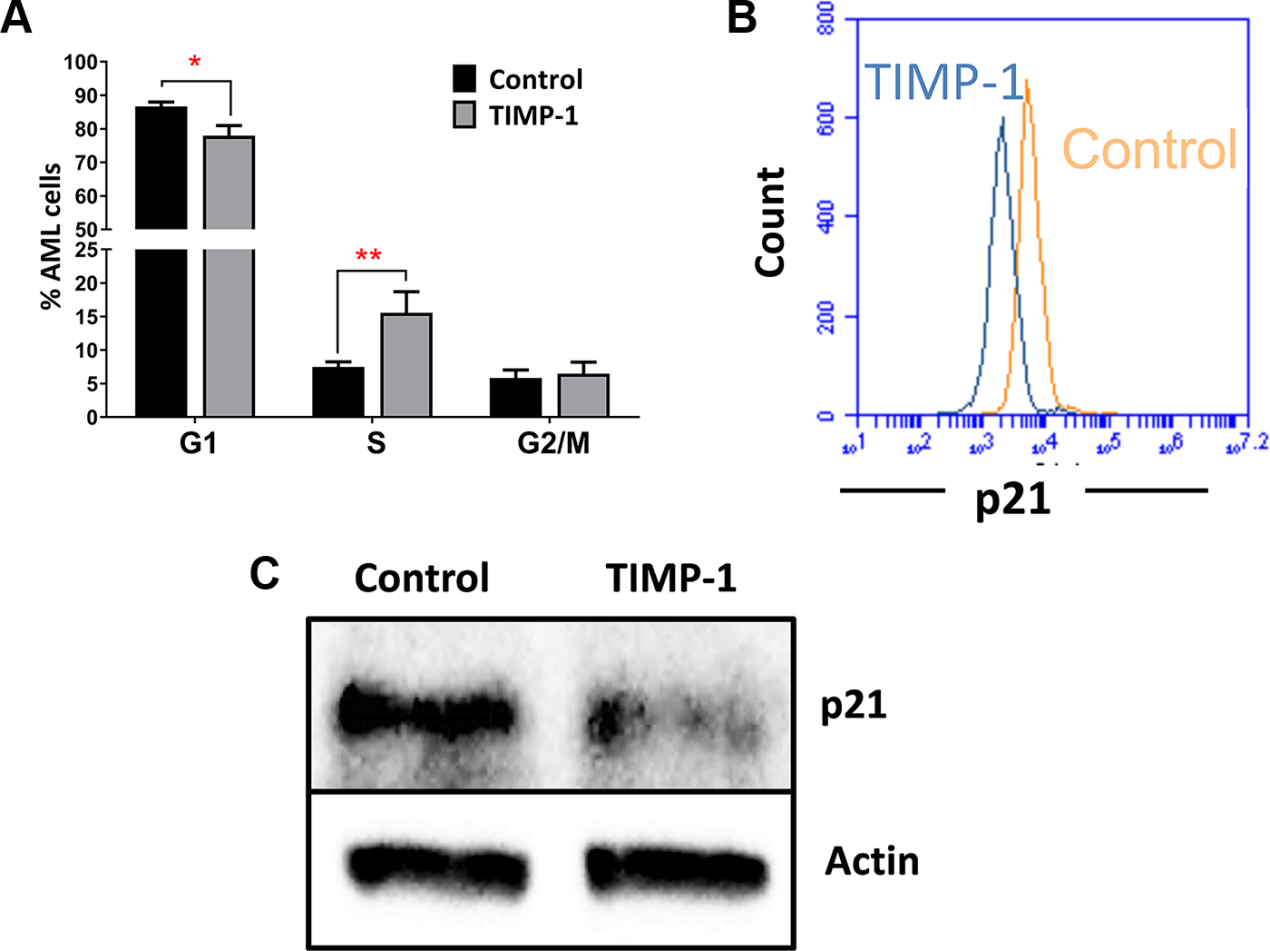
TIMP-1 promotes cell cycle of leukemic blasts (**A**) Results are expressed as the percentage of cells in different phases of the cell cycle. TIMP-1 promote cell cycling of leukemic blasts cells from AML patients (phase G1 *p ≤ 0.05 and phase S **p ≤ 0,01; *n* = 9). Their distribution in the different phases of cell cycle was assessed by PI staining after 24 hrs. Data are presented as mean ± SEM. (**B**) Representative histogram of p21^WAF1/CIP1^ of leukemic blasts after exposure to TIMP-1 for 24 hrs. The downregulation of cyclin-dependent kinase inhibitor p21^WAF1/CIP1^, which promotes cell cycle progression, were measured by flow cytometry as MFI, and is shown only in the presence of TIMP-1 (MFI: 2275 ± 524 TIMP-1 treated cells vs 3110 ± 223,1 untreated cells; *n* = 12). Specifically, only 2 patients (PT #11 and #15 in the Table patients) were not responsive to TIMP-1 treatment in p21 downregulation. (**C**) AML cells were incubated with TIMP-1 for 24 hrs and the relative level of p21 protein was determined by western blotting (*n* = 2). Representative western blot of one AML patient. β-actin was used as a loading control.

We then studied the effect of TIMP-1 on cycling of AML cells. As shown in Figure 3A, after 24 hours of TIMP-1 incubation, we observed a reduced percentage of cells in G0/G1 phase (percentage of untreated G0/G1-phase cells 86.66 ± 1.34 % vs 77 ± 3.03 % of TIMP-1-treated cells; *p ≤* 0.05). Simultaneously, we detected a slight, but significant, increase in the percentage of leukemic cells in S-phase (percentage of untreated S-phase cells 7.4 ± 0.77 % vs 15.5 ± 3.1% of TIMP-1-treated cells; *p ≤* 0.01). These data suggested a role of TIMP-1 in promoting AML cell cycle progression. In order to confirm this observation, we analyzed the effect of TIMP-1 on expression of p21^WAF1/CIP1^, a cell cycle inhibitor [33] (Figure 3B). Interestingly, the normalized mean fluorescence intensity (nMFI) of p21 expression was significantly reduced in leukemic cells after exposure to TIMP-1 for 24 hours as compared with untreated cells (nMFI: 3.1±1.1 vs 4.5±1.5, respectively; *p ≤* 0.05; data not shown). To further confirm our results, we analysed p21 protein reduction by western blot (Figure 3C). This assay confirmed the reduction of p21 protein after treatment with TIMP-1, whereas transcript levels were not affected (data not shown). These results suggest that TIMP-1 stimulates cell-cycle progression of leukemic blasts from AML patients.

### TIMP-1 enhances CXCL12-driven migration of AML blasts

**Figure 4:**
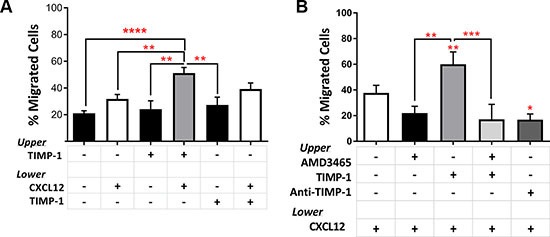
Pre-treatment with TIMP-1 significantly promotes migration of AML cells (**A**) AML cells were seeded in the upper compartment of a chamber Transwell migration assay ± TIMP-1 (100 ng/ml) (*n* = 18). TIMP-1 was added to the bottom compartment with or without CXCL12 (100 ng/ml), whereas was used alone in the top compartment. After over-night at 37°C, cells in the bottom compartments were counted and percentage of migrated cells calculated. (**p ≤ 0.01; ****p ≤ 0.0001; *n* = 18). The mean number of migrated cells was 13031 ± 2161 for spontaneous migration, 15410 ± 2367 for migration toward CXCL12 gradient, 23581 ± 3204 in the presence of TIMP-1 toward CXCL12 gradient and 22070 ± 4050 in the presence of TIMP-1 and CXCL12 as chemo-attractants. (**B**) Migrated AML-derived leukemic blasts toward CXCL12 gradient after pre-treatment with AMD3465 (CXCR4 antagonist) for 30 min ± TIMP-1 (*n* = 9) or anti-TIMP-1 neutralizing Ab (5 μg/ml; *n* = 3) to evaluate the effects on migratory rate (*p ≤ 0.05; **p ≤ 0.01; ***p ≤ 0.001). Data are presented as mean ± SEM.

To test the effect of TIMP-1 on the migratory capacity of AML cells, we assessed, *in vitro*, the response of AML cells toward a CXCL12 gradient in presence or absence of TIMP-1. As reported in Figure 4A, the migration rate of AML cells, when TIMP-1 was added in absence of CXCL12, was not significantly higher than control samples. Although these results could rely, at least in part, on TIMP-1's survival effects, the addition of TIMP-1, in presence of CXCL12, increased the migration rate of leukemic cells over that observed with medium (spontaneous migration) and CXCL12 alone (*p ≤* 0.0001 and *p ≤* 0.01, respectively). Conversely, when TIMP-1 was added to CXCL12 as chemo-attractant, the migration of AML cells was not significantly increased. No differences in spontaneous or CXCL12-induced migration were observed when PB or BM cells were compared (data not shown). However, migration of TIMP-1 treated PB cells toward CXCL12 gradient was slightly higher than the migration of the BM cells (PB vs BM, 57.14% ± 5.5% vs 46.09% ± 7.7%, respectively; p=ns). To further support this finding, we investigated the migratory response of AML cells after pre-incubation with AMD3465, a potent and selective CXCR4 antagonist and anti-TIMP-1 neutralizing Ab. After incubation with AMD3465, the migration of leukemic blasts was inhibited and the effect of CXCL12 was comparable to medium alone (Figure 4B). Importantly, pre-incubation with the CXCR4 inhibitor completely abrogated the effect of TIMP-1 treatment toward CXCL12-gradient (*p ≤* 0.001). Also, anti-TIMP -1 neutralizing Ab reversed the effects of TIMP-1 on migratory response of AML cells.

### CD63 is required for TIMP-1-mediated effects on AML blasts

**Figure 5:**
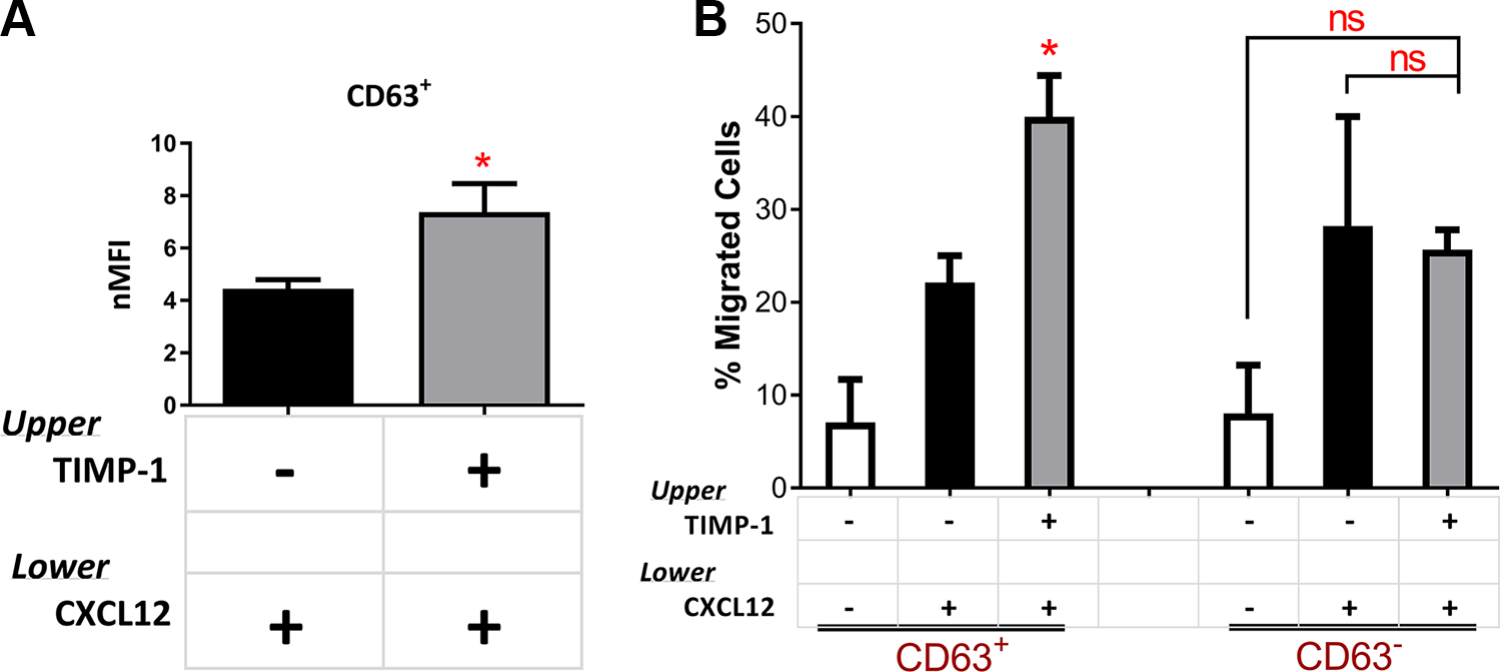
TIMP-1's effects on migration are mediated by the CD63 tetraspanin receptor (**A**) AML cells were seeded in the upper compartment of a chamber transwell migration assay ±TIMP-1 (100 ng/ml). After 24 hrs migrated AML-derived leukemic blasts toward CXCL12 gradient after pre-treatment with TIMP-1 show increased expression of TIMP-1 receptor (CD63) as compared with untreated cells. Results are expressed as normalized Mean Fluorescence Intensity (nMFI). (*p ≤ 0.05; *n* = 10). (**B**) Based on CD63 expression, leukemic blasts were sorted in two separate fractions, CD63^–^ and CD63^+^, and their capability to respond to TIMP-1 stimulation was assessed by migration assay. AML cell fractioning were seeded in the upper compartment of a chamber Transwell migration assay ± TIMP-1 (100 ng/ml) toward CXCL12 (*p ≤ 0.05; *n* = 3). The mean number of migrated CD63^+^ cells was 705 ± 46 for spontaneous migration, 2213 ± 287 for migration toward CXCL12 gradient, 3720 ± 600 in the presence of TIMP-1 toward CXCL12 gradient. The mean number of migrated CD63^–^cells was 805 ± 52 for spontaneous migration, 2695 ± 555 for migration toward CXCL12 gradient, 2575 ± 370 in the presence of TIMP-1 toward CXCL12 gradient. Data are presented as mean ± SEM.

To elucidate the molecular pathway ignited by TIMP-1 and its cytokine-like activity in leukemic blasts, we first investigated the expression and the functionality of CD63, which is known to mediate TIMP-1 effect [16]. The mean percentage of CD63-positive blasts from the total of AML patients was 60.83% ± 5.485% (range, 26.40–93.40). In particular, the mean percentage was 64.05% ± 8.024% (range, 32.1–93.4) in PB samples and 56.53% ± 7.476% (range, 26.4–79.6) in BM samples. Of note, we did not find any significant correlation between patient characteristics and CD63 expression or different functions mediated by TIMP-1 (data not shown). Interestingly, migrated leukemic blasts toward CXCL12 plus TIMP-1 had a significantly increased expression of CD63 as compared to the cells migrated toward CXCL12 alone (*p ≤* 0.05) (Figure 5A). We then, sorted CD63 positive and negative leukemic blasts and tested their ability to respond to TIMP-1 in migration assay. Noteworthy, only the migratory capacity of CD63^+^ fraction appeared significantly promoted (*p ≤* 0.05; Figure 5B), while the proportion of migrating CD63^–^ AML cells in presence of TIMP-1 cells was comparable to that migrated with CXCL12-gradient alone. Overall, these findings support the hypothesis that TIMP-1 modulates the CXCL12-mediated migration through the interaction with the CD63 receptor, exerting cytokine-like function in leukemic blasts.

### TIMP-1 induced PI3K/Akt and HIF-1α signaling pathway in leukemic blasts

**Figure 6:**
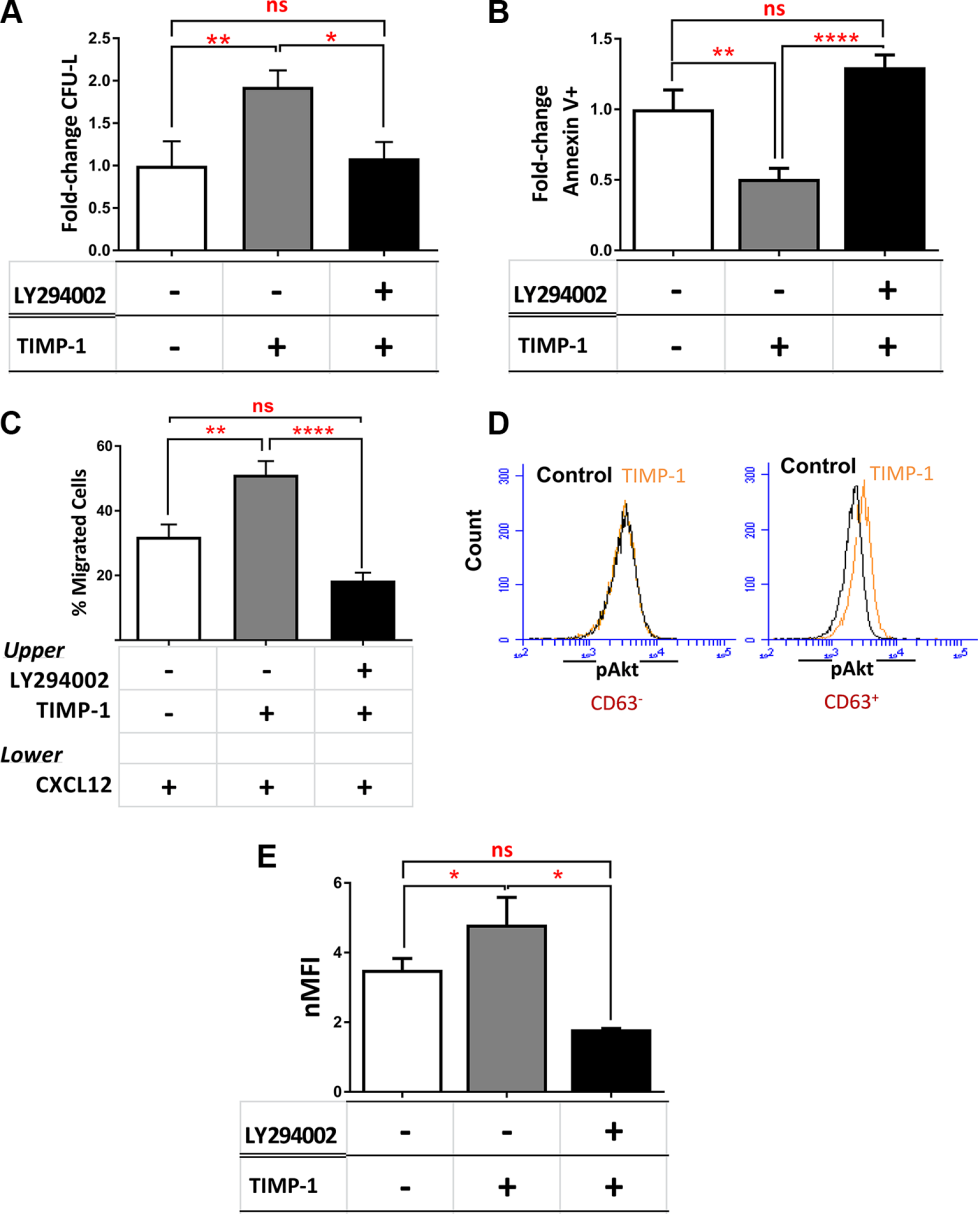
TIMP-1 effects in AML cells are mediated by PI3K/Akt and HIF-1α signaling axis (**A**) AML cells were cultured in the presence or absence of TIMP-1 (100 ng/mL) after pre-treatment with LY294002 (20 μM) and the correspondent clonogenic output was assessed after 14 days of culture (*p ≤ 0.05; **p ≤ 0.01; *n* = 3). The mean number of CFU-L was 19.7 ± 5.6, 30.3 ± 9.6 and 14.0 ± 6.2 for control, TIMP-1-treated and LY294002-pre-treated AML cells, respectively. The results are expressed as growth fold-change versus untreated control samples. (**B**–**C**) In the same culture condition we tested apoptosis after 24 hrs (*n* = 8) and migratory behaviour toward CXCL12 gradient (**p ≤ 0.01; ****p ≤ 0.0001; *n* = 8). The mean numbers of migrated cells were 9032 ± 2970, 16432 ± 3881 and 8450 ± 1638 for control, TIMP-1-treated and LY294002-pre-treated AML cells, respectively. The results are expressed as fold-change versus untreated control samples and as mean of migrated cells, respectively. (**D**) Representative panel of pAkt levels in TIMP-1-treated versus untreated cells of CD63^–^ leukemic blasts (left panel) or CD63^+^ leukemic blasts (right) (*n* = 3). (**E**) Level of Akt phosphorylation (Thr308) in total AML cells exposed for 15 minutes to TIMP-1 (100 ng/mL) or after pre-treated with LY294002, as assessed by flow cytometry. Results are expressed as normalized MFI: 3.5 ± 0.32 (MFI 1387 ± 264) untreated cells vs 4.8 ± 0.7 (MFI 1524 ± 274) TIMP-1 treated cells (*p ≤ 0.05; *n* = 13). Specifically, only 3 patients (PT #4-#7-#8 in the Table patients) were not responsive to TIMP-1 treatment in pAkt activation (*p ≤ 0.05; *n* = 13).

We then investigated the signaling pathway activated by TIMP-1 exposure. We previously demonstrated that in normal CD34^+^ cells PI3K/Akt is the main signaling pathway induced by TIMP-1 [22]. Based on these results, we tested TIMP-1 effects on AML cell function in presence of the PI3K-inhibitor LY294002. Pre-incubation of AML cells with LY294002 plus TIMP-1 significantly decreased their clonogenic output as compared to TIMP-1 alone (*p ≤* 0.05) (Figure 6A), reverted TIMP-1-mediated anti-apoptotic activity (*p ≤* 0.0001) (Figure 6B) and its migratory capacity on AML cells (Figure 6C) (*p ≤* 0.0001). These results suggest that, similarly to normal HSC, in leukemic blasts the pathway of PI3K/Akt may be involved in the signaling induced by TIMP-1. To further support this hypothesis, we examined the activation/phosphorylation of Akt in TIMP-1-treated AML cells. We firstly observed that the addition of TIMP-1 resulted in pAkt-Thr308 phosphorylation of CD63^+^, but not CD63^–^, AML cells (Figure 6D), confirming that CD63 binding may be important to pAkt activation by TIMP-1. Next, we assessed by flow cytometry the levels of Akt phosphorylation in leukemic blasts after exposure to TIMP-1. As indicated in Figure 6E, the mean value of nMFI of phospho-Akt was 3.5 ± 0.32 in untreated cells vs 4.8 ± 0.7 after TIMP-1 treatment (*p ≤* 0.05). Moreover, we determined the levels of pAkt upon TIMP-1 treatment in AML cells by Western blotting (Supplementary Figure S3). As expected, after PI3K inhibition the treatment with TIMP-1 had no effect on Akt phosphorylation. Finally, we examined the effect of TIMP-1 on the expression of HIF-1α, described as a downstream pathway following PI3K/Akt activation [34]. Of note, the expression of HIF-1α mRNA in leukemic blasts was found to be modulated by TIMP-1 stimulation. Specifically, after 24 hours in presence of TIMP-1, a significant increase in the expression of HIF-1α was observed (*p ≤* 0.05; Supplementary Figure S4). Together, these data support the hypothesis that TIMP-1 modulates the function of leukemic blasts via PI3K/Akt and HIF-1α axis.

### The leukemic BM microenvironment enhances the effects of TIMP-1 on migration of leukemic blasts

**Figure 7:**
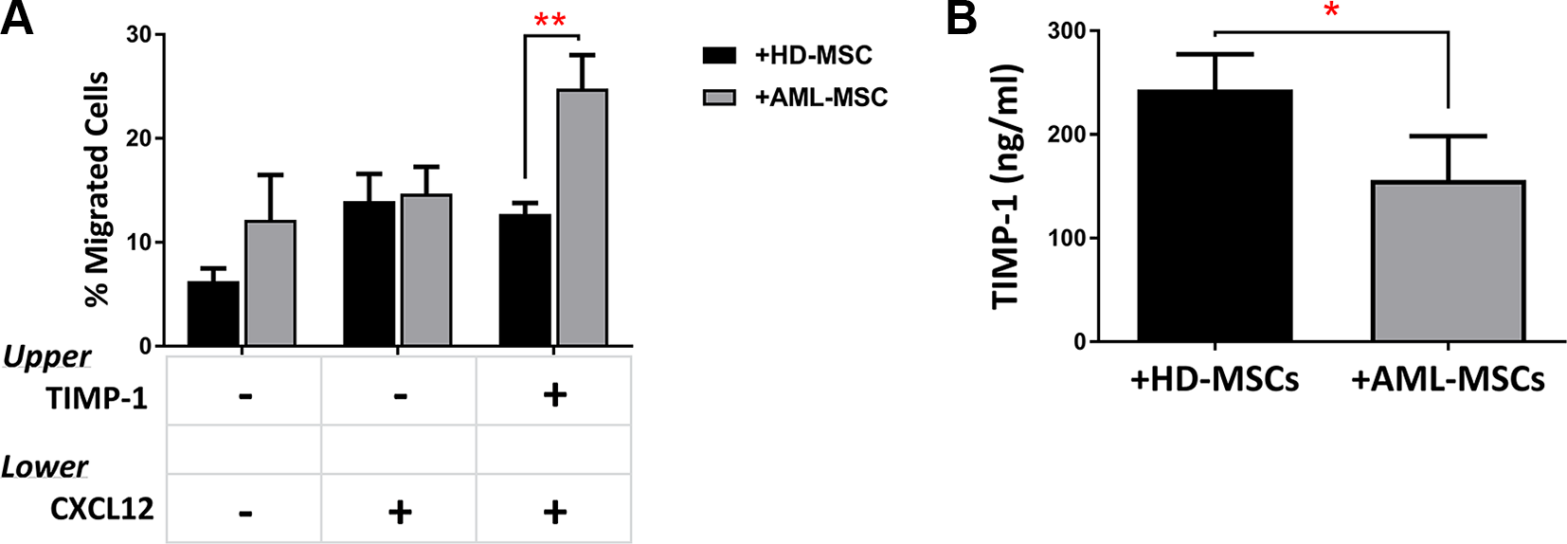
TIMP-1's effects on BM microenvironment: TIMP-1 significantly promotes migration of AML-derived leukemic cells after co-culture with AML MSCs AML cells were co-cultured with normal (*n* = 2) or leukemic MSCs (*n* = 3) for 3 days ± TIMP-1 (100 ng/ml). After co-culture, AML cells were collected, counted and seeded in the upper compartment of a chamber Transwell migration assay toward CXCL12-gradient (100 ng/ml). (**A**) The addition of TIMP-1 for 3 days in the presence of leukemic MSCs affects the migratory behaviour of AML-derived blasts cells toward CXCL12 alone (**p ≤ 0.01) (*n* = 6). The mean numbers of migrated cells were 7784 ± 2794 for spontaneous migration, 7315 ± 1776 for migration CXCL12-driven and 17732 ± 6735 for migration CXCL12-driven after TIMP-1 treatment after co-cultures with AML-MSCs. On the contrary the mean numbers of migrated cells were 2730 ± 770 for spontaneous migration, 6223 ± 1811 for migration CXCL12-driven and 5613 ± 1167 for migration CXCL12-driven after TIMP-1 treatment after co-cultures with HD-MSCs. (**B**) Leukemic cells were co-cultured in presence of a normal or leukemic stroma layer. After 3 days of co-culture, supernatants were collected and analysed for TIMP-1 level using ELISA (*p < 0.05; *n* = 3). Data are mean ± SEM. “HD” means healthy donor.

It is known that migration of AML cells is regulated by stromal cells, such as MSCs, producing CXCL12 [35, 36]. To further investigate the role of TIMP-1 within BM microenvironment of AML patients, we tested the migratory ability of leukemic blasts after co-cultures with normal or leukemic MSCs in presence or absence of TIMP-1. No significant biological difference was found between normal and AML-MSCs in terms of phenotype and differentiation capacity (data not shown). As shown in Figure 7A, the CXCL12-driven migration of leukemic blasts was not affected by TIMP-1 pre-incubation in co-cultures with normal MSCs. Conversely, when leukemic blasts were co-cultured with AML-MSCs, the addition of TIMP-1 resulted in increased migration rate as compared to that observed in presence of normal MSCs (*p ≤* 0.01). Interestingly, when we analyzed the levels of TIMP-1 in the supernatants of co-cultures of AML cells with leukemic or normal MSCs, we found that TIMP-1 level was higher in presence of HD-MSCs over AML-MSCs (263 ± 19.68 ng/ml and 162 ± 24.43 ng/ml, respectively; *p ≤* 0.05; Figure 7B).

### TIMP-1 enhances leukemic cell survival and proliferation in presence of leukemic MSCs

**Figure 8:**
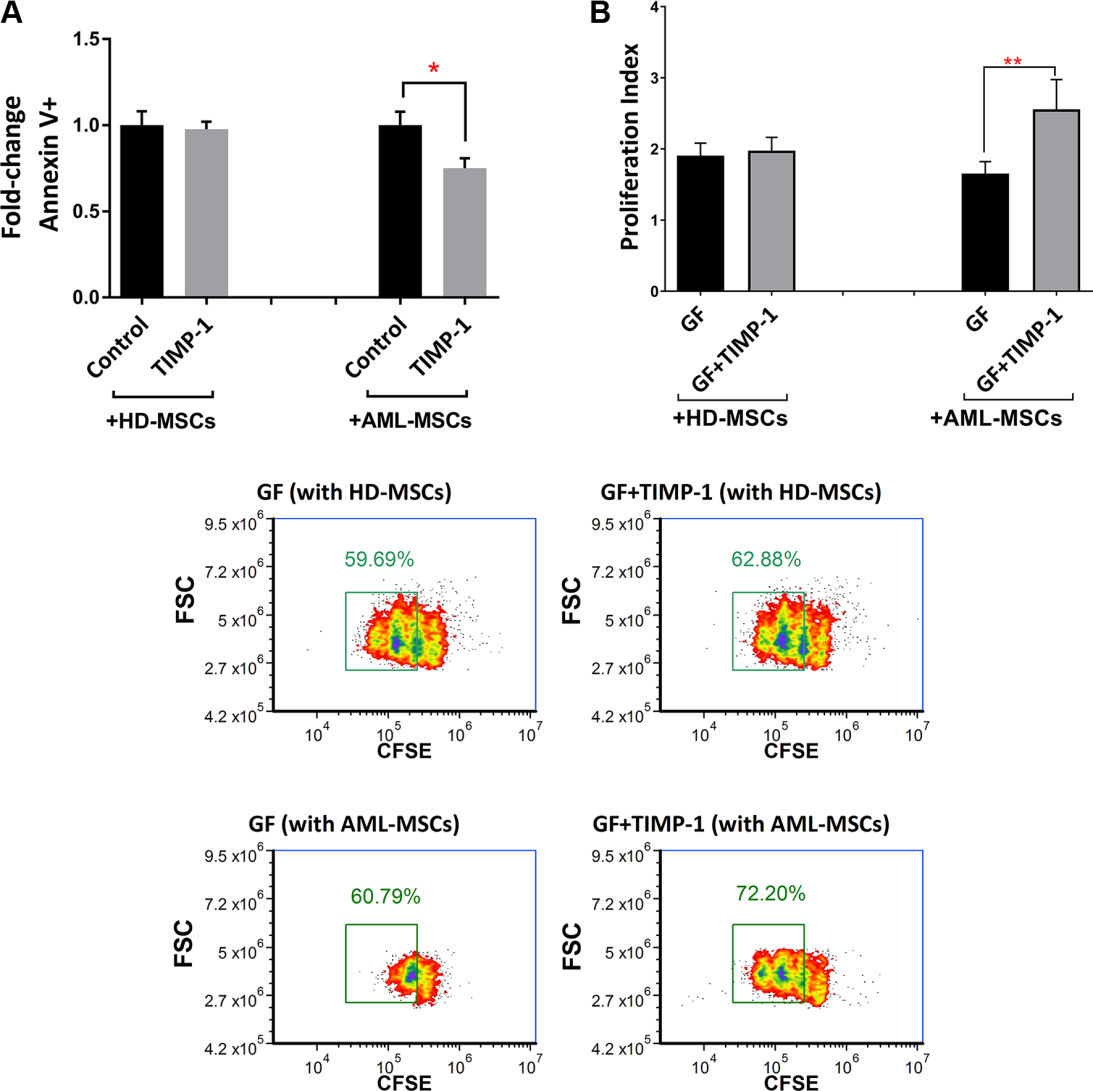
TIMP-1's effects on BM microenvironment: TIMP-1 promotes cell survival and proliferation in AML-derived blasts in the presence of AML-MSCs (**A**) AML cells were co-cultured with normal (*n* = 2) or leukemic MSCs (*n* = 3) for 3 days ± TIMP-1 (100 ng/ml) to test survival of leukemic cells (*p < 0.05; *n* = 4). (**B**) AML cells were co-cultured with normal or leukemic MSCs for 5 days with growth factors ± TIMP-1 (100 ng/ml). Proliferation index of CFSE-labeled leukemic blasts was calculated. Cells maintained in culture in the presence of medium alone was evaluated as 1 of proliferation index. AML MSCs displayed, at culture day 5, a significantly increased proliferation index, as compared to leukemic cells co-coltured with normal MSCs (**p ≤ 0.01; *n* = 12). (**C**) Representative dot-plots showing the percentage of proliferative cells gated with reduced CFSE content by flow cytometry. Left panels, AML cells were co-cultured with normal (on the top) or leukemic MSCs (on the bottom) for 5 days with growth factors only. Right panels, AML cells were co-cultured with normal (on the top) or leukemic MSCs (on the bottom) for 5 days with growth factors ± TIMP-1. Data are mean ± SEM. GF, growth factors.

Recently, several studies have demonstrated the essential role of BM stromal-derived factors in regulation AML cell proliferation, thus conferring chemo-resistance to leukemic cells [2, 11]. To test the differential effect of TIMP-1 on AML cell survival in presence of normal versus leukemic MSCs, co-cultures were set up in the presence or absence of TIMP-1. As shown in Figure 8A, the survival of leukemic blasts was not affected by TIMP-1 in co-cultures with normal MSCs. However, TIMP-1 showed a slight reduction in apoptotic rate in co-culture with AML-MSC (*p ≤* 0.05). Moreover, AML cell proliferation was evaluated with normal MSCs or AML-MSCs in the presence or absence of TIMP-1 plus growth factors, thus mimicking the BM microenvironment. When AML cells were co-cultured with normal MSCs, cell proliferation was not significantly modified after exposure to TIMP-1. By contrast, in co-cultures with AML-MSCs, the combination of TIMP-1 plus growth factors resulted in highly significant increase of the proliferation index as compared with blasts co-cultured with growth factors alone (*p ≤* 0.01; Figure 8B–8C). In addition, a non-significant trend to increased cell proliferation was found from CD63-positive fractions in AML cells (data not shown).

Taken together, the results reported in Figures 7 and 8 support the notion that leukemic microenvironment is deeply dysregulated in AML and TIMP-1 may preferentially exert its effects on leukemic cells in presence of leukemia-derived MSCs.

## DISCUSSION

Here we demonstrate that TIMP-1 increases the clonogenic efficiency, the survival and the migratory capacity of AML blasts by binding to CD63 receptor which, in turn, results in the activation of PI3K, Akt phosphorylation and regulation of downstream targets, such as p21 and HIF-1α. Moreover, we showed the potential role of TIMP-1 in the interplay between leukemic blasts and AML MSCs. Together, these findings suggest that TIMP-1 displays cytokine-like features in the BM microenvironment of AML patients, suggesting a link between the inflammatory microenvironment and leukemogenesis.

Several papers report that the activation of PI3K/Akt signaling is associated with poor outcome in hematological malignancies and this pathway is crucial to cancer cell survival and growth [37]. Here we demonstrate that, similarly to CB-derived CD34^+^ [22], binding of TIMP-1 to CD63 receptor is instrumental for modulation of AML cell functions. Whereas the role of CD63 in regulating cancer cell functions, such as cell activation, adhesion, differentiation and migration, is well-established in solid tumors [16, 19], its role remain unclear as for hematological disease. Schubert et al. [38] showed higher CD63 expression in AML-long-term surviving capacity (LTSC) growth in NOD/SCID mice, hinting the potential role in cell adhesion and motility. In the present paper we demonstrate that about half of leukemic blasts are CD63 positive. At the functional level, binding to CD63 is required for TIMP-1-mediated main effects on AML blasts along the PI3K/Akt pathway, as previously observed in CB CD34^+^ cells. In addition, we found that TIMP-1 stimulates the cell-cycle progression of leukemic blasts with a subsequent down-regulation of the cell-cycle inhibitor p21. Similarly to other pro-inflammatory cytokines (e.g. IFN-γ and IL-4), TIMP-1 was shown to be an inducer of HIF-1α expression in a liver metastasis model [39]. Moreover, HIF-1α mRNA and protein are overexpressed in human cancer and leukemic stem cells [40–41]. Our data suggest, for the first time, that upregulation of HIF-1α mRNA may be mediated by TIMP-1 in AML.

Leukemic blasts have been shown to have higher levels of TIMP-1 transcripts and increased TIMP-1 expression was also observed in Non-Hodgkin lymphomas and Burkitt B-cell lymphoma cell lines [28–30]. Accordingly [32], in the present work we found that the BM plasma levels of TIMP-1 are detectable in AML patients. At variance from normal CB-derived CD34^+^ cells, where TIMP-1 was shown to affect only the clonogenic potential, in AML TIMP-1 exerts additional effects, such as improving cell survival and CXCL12-mediated migration. In the leukemic microenvironment, CXCL12 is secreted both by BM stromal and AML cells and critically modulates cell survival and retention of LSCs within BM [43, 44]. Our demonstration that TIMP-1 pre-treatment of leukemic blasts promotes their migration towards CXCL12 support the finding of high levels of TIMP-1 in the BM of AML patients, suggesting a role for this molecule in promoting the migration of leukemic blasts and their dissemination in extramedullary site.

The dynamic interplay between leukemic cells and stromal cells is a crucial aspect of AML [45]. It is clear that leukemic cells alter their BM microenvironment to support leukemic hematopoiesis while disrupting normal HSC homeostasis [46]. This interaction via cytokines, chemokines and adhesion molecules is responsible for reduced chemo-sensitivity of leukemic sub-clones [47]. By contrast, the potential role of TIMP-1 in the interaction of leukemic cells with BM stromal cells is mostly unknown. Of note, previous investigations demonstrated that among 23 cytokines differentially expressed, at the molecular level, between normal and leukemic BM, only TIMP-1 was confirmed at the protein level suggesting a role in leukemia initiation and progression [32]. Moreover, the crosstalk between the matrix metalloproteinases system and chemokines network may modulate different regulators of cytokine release by primary human AML cells (e.g. NF-kB) [14]. In this view, our functional data support a role of TIMP-1 as an adverse factor within leukemic microenvironment. Interestingly, TIMP-1 has been shown to preferentially increase AML blasts survival, proliferation and migration in presence of AML-MSCs. Such discrepancy in comparison to normal MSCs is far to be completely elucidated. A possible explanation is that secreted TIMP-1 from normal MSCs saturate potential TIMP-1 receptors, reverting and masking the pro-survival and pro-migratory effects of exogenous TIMP-1. Indeed, previous studies demonstrated the different production of TIMP-1 by normal human MSCs (hMSCs), AML long-term marrow cultures (LTMC) and leukemic BM [32, 48, 49]. Although limitation of the present study is the small sample size of patients, our findings point to an abnormal interplay between leukemic cells and their microenvironment and suggest that AML stroma may be a novel target to prevent the development of leukemia.

In conclusion, according to the “bad seeds in bad soil” concept [50], our data provide new evidence for TIMP-1 as a ‘bad’ linker between inflammation and leukemogenesis. Our preclinical results may provide the biological background for further investigating TIMP-1 as a potential therapeutic target in the context of leukemic microenvironment.

## MATERIALS AND METHODS

### Primary samples

Peripheral blood (PB, *n* = 9) and bone marrow (BM, *n* = 9) samples were obtained from AML patients (*n* = 18) at diagnosis after informed consent was signed. The percentage of AML blasts was always > 90%. Patient characteristics are summarized in Supplementary Table S1. As control samples, buffy-coats from blood transfusion processing and BM samples from patients with lymphomas, undergoing staging, with no sign of BM involvement were used. Control samples were rendered anonymous and not referred to individuals. Mononuclear cells (MNCs) were separated by stratification on Lympholyte-H 1.077 g/cm^3^ gradient (Gibco-Invitrogen, Milan, Italy), followed by red blood cell lysis for 15 min at 4°C. Briefly, AML blasts were cultured in RPMI 1640 (Thermo Fisher Scientific,Waltham, MA), supplemented with 10% fetal calf serum (Thermo Fisher Scientific, Waltham, MA). Mesenchymal stromal cells (MSCs) were obtained from BM of healthy donors (*n* = 2) or AML patients (*n* = 3) as previously described [31]. MSCs were cultered at a density of 5,000 to 10,000 cell/cm^2^ in DMEM (Lonza, Veriers, Belgium) supplemented with 10% FBS. The isolated MSCs at passage 3 were evaluated by flow cytometric analysis for immunophenotype and were used for co-culture experiments (passage 3 to 5) after irradiation (10,000 Gy).

### Measurement of TIMP-1 levels

Serum was obtained from PB and plasma from BM samples of AML patients at diagnosis and stored at −80°C. The concentration of TIMP-1 in the supernatants from primary AML cells co-cultured with MSCs and the serum/plasma samples have been analysed by an ELISA assay (Boster Biological Technology Co., Pleasanton, CA) according to manufacturing instruction.

### Colony-forming unit (CFU) assays

AML cells were cultured in methylcellulose medium (human StemMACS HSC-CFU lite w/ Epo, Miltenyi Biotech, Germany) at 1–5 × 10^5^cells/mL in 35-mm Petri dishes in the presence or absence of TIMP-1 (10–300 ng/mL, Thermo Scientific, Pierce Biotechnology, Rockford, IL, USA). Cell cultures were maintained at 37°C in a fully humidified atmosphere with 5% CO_2_ and after 14 days CFU-Ls were scored. The experiments were performed in duplicates plates and a colony was considered to be an aggregate of 20 and more cells.

### Apoptosis assay

AML cells were cultured in the presence of TIMP-1 (100 ng/mL) and the apoptotic rate was evaluated at different time points by AnnexinV/Propidium Iodide Staining (Annexin-V-FLUOS-kit,Roche, Penzberg, Germany). Sample acquisition and analysis was performed on a BD Accuri C6 flow cytometer (BD Biosciences). Where indicated, cells were cultured in the presence of LY294002 (PI3K inhibitor). Cell viability was also measured by CellTiter 96 Aqueous One Solution (Promega). AML cells were cultured in presence of TIMP-1 (100 ng/mL) for 24–48 hours and cell cycle distribution were evaluated at different time points. Treated cells were first permeabilized with NP-40 (15 min at RT) and then labelled with propidium iodide (PI)/RNAse staining kit (BD Bioscience) (15 min at RT, in the dark). The DNA content was assessed by BD Accuri^TM^C6 (BD Bioscience) and results were analyzed by FCS express 4 software.

### Cell sorting and flow cytometry

Flow cytometry studies were performed as previously described [22]. Leukemic blasts were labelled with an anti-CD63 antibody phycoerythrin (PE)-conjugated (eBioscience, San Diego, CA) and sorted on a FACS Aria (Becton Dickinson, BD Bioscience, San Jose, CA).

Sorting gates were carefully drawn in order to avoid any cross-contamination among the two populations. Purity checks by flow cytometry was performed on each isolated cell population and the mean purity was 90% ± 5%.

Phospho-Akt intracellular staining was performed as previously described [22]. Leukemic blasts were treated with TIMP-1 (100 ng/ml) for 3.5–15 min at 37°C, 5% CO_2_. After fixation in 4% paraformaldehyde (PFA) in PBS/permeabilization, cells were washed with phosphate-buffered saline (PBS), 0.1% bovine serum albumin (BSA) and incubated with pAkt (Thr308) (C31E5E) Rabbit monoclonal antibody (PE-conjugate; Cell Signaling) for 30 min at room temperature (RT) in the dark. Data were analyzed using FCS express 4 Flow Cytometry analysis software (De Novo Software, Glendale, CA, USA).

For intracellular analysis of p21 protein, leukemic blasts were treated with TIMP-1 (100 ng/ml) for 24 hours at 37°C, 5% CO_2_. After fixation in 4% PFA, 0.1% Saponin was used to permeabilize the cells. As above cells were incubated with p21 antibody or negative control antibody and analysed by flow cytometry.

Where indicated, cells were cultured in the presence of 20 μM PI3K inhibitor (LY294002) (Sigma-Aldrich, Saint Louis, MO, USA) or 5 μM CXCR4 antagonist (AMD3465) for 30 minutes (Abcam, Cambridge, UK), followed by exposure to TIMP-1 (100 ng/ml). In selected experiments, cells were cultured in the presence of anti-TIMP1 antibody (5 μg/ml) (ab77847, Abcam, Cambridge, UK).

Negative controls were isotype-matched irrelevant MoAbs from BD Pharmingen or eBioscience and were used for setting limits of nonspecific immunoglobulin cell binding. Specifically, the following MoAbs were used: APC or PE Mouse IgG1, κ Isotype Control (Clone MOPC-21) and PE-Cyanine7 or FITC IgG1, k Isotype Control (Clone P3.6.2.8.1).

In order to normalize our data for p21 and pAkt staining, we calculated the normalized MFI (nMFI), as MFI of the stained sample/MFI of the negative control sample.

### CFSE labelling and analysis

Leukemic proliferation was monitored by flow cytometry, monitoring Green fluorochrome carboxyl fluorescein diacetate succinimidyl ester (CFSE, Molecular Probes Europe, Leiden). AML cells were labelled with CFSE (5 μM) for 4 min at RT in PBS, 0.1% BSA, followed by the addition of ice-cold RPMI with 10% FBS to prevent further dye uptake. Cells were washed 3 times in ice-cold medium and maintained in culture for 5 days in RPMI with 10% FBS, with or without TIMP-1 (100 ng/ml) supplemented with growth factors (GF): stem cell factor (SCF; 50 ng/mL, Amgen, Thousand Oaks, *CA*), interleukin (IL)-3 (50 ng/mL, Miltenyi Biotech, BO, Italy), granulocyte macrophage colony-stimulating factor (GM-CSF; 10 ng/ml, Peprotech, London, UK). Data were analyzed by FCS express 4 Flow Cytometry analysis software.

### Migration assay

Migration of leukemic blasts was assayed towards a CXCL12 gradient (100 ng/mL, Meridian Life Science,Memphis, TN) in trans-well chambers (diameter 6.5 mm, pore size 8 μm; Costar; Corning, New York, USA). Briefly, 50 μl of RPMI 1640 plus 10% FBS containing 0,5x10^5^ cells pre-incubated or not with TIMP-1 were added to the upper chamber and 150 μl of medium ± CXCL12 were added to the bottom chamber. After overnight incubation at 37°C in 5% humidified CO_2_ atmosphere, inserts (upper chambers) were removed and transmigrated cells were counted by Trypan Blue exclusion test in a Neubauer chamber. The amount of migrated cells was expressed as a percentage of the input, applying the following formula: (number of cells recovered from the lower compartment/total number of cells loaded in the upper compartment) × 100%.

### Co-culture experiments

AML cells were cultured with MSCs at ratio 1:10 in a direct cell-to-cell contact co-cultures. After 3 days in presence or absence of TIMP-1 (100 ng/ml) the migratory behaviour of leukemic blasts was assessed by migration assay toward CXCL12 gradient. After 5 days with SCF (50 ng/mL), IL-3 (50 ng/mL), GM-CSF (10 ng/ml) ± TIMP-1 proliferation was evaluated by flow cytometry, monitoring CFSE labelling. After separation, MSC monolayers were examined by microscopy to confirm that the monolayer was not damaged. To verify lack of significant contamination in collected AML cells, the expression of CD45 was measured.

### Western blot analysis

AML cells were collected by centrifugation, washed with PBS 1% Phenylmethanesulfonyl fluoride PMSF (Sigma-Aldrich) and total protein extracts were separated by sodium dodecyl sulfate polyacrylamide gel electrophoresis, transferred onto nitrocellulose membrane (GE Healthcare, Buckinghamshire, UK), and then subjected to Western blotting. Membranes were saturated for 1 hour at room temperature in blocking buffer (1X tris-buffered saline, 5 M NaCl, 20 mM Tris-HCl; pH 8.0, 0.1% Tween-20, 4% BSA) and then incubated overnight at 4°C with the specific primary antibodies: rabbit anti-pAkt Thr308 [1:3000] (18F.H11; Abcam) or p21 [1:1000] (#2947, Cell Signaling) in 4% BSA in TTBS (TBS 0,1% Tween-20) for 15–20 h at 4°C. Membranes were washed three times for 5 min in TTBS and secondary antibodies (donkey anti-rabbit HRP (sc-2313), donkey anti-mouse HRP (sc-2314) [1:40000] (Santa Cruz Biotecnology) were added for 1h at room temperature, and then membrane-bound were washed three times for 5 min in TTBS as described previously. Signal intensities in single blots were measured by means of ChemiDoc-It instrument equipped with a dedicated software (Launch VIsionWorksLS, Euroclone). Protein expression was quantified by band densitometric analysis using IMAGEJ 1.44p Launcher software (National Institutes of Health, Bethesda, MD, USA).

### RNA extraction and real-time polymerase chain reaction (Real-Time PCR) analysis

Total RNA was isolated from treated cells using RNAeasy Micro Plus Kit (Qiagen, MI, Italy). First-strand synthesis was performed with Improm2 (Promega,Madison, WI) and Real Time PCR was performed with Taqman probe sets (Applied Biosystems, Foster City, CA) on a ABI Prism 7700 Sequence Detector for 40 cycles. Primer sequence used for HIF-1α or p21. A human internal control glyceraldehyde-3-phosphate dehydrogenase (GAPDH) was included in every reaction for normalization and expression was measured for each assay relative to the GAPDH internal standard (ΔCt). The fold change was calculated from the formula 2^ΔΔCt^.

### Statistical analysis

The results are expressed as the mean ± SEM. Differences between groups were compared by using either a Student's *t-test* or ANOVA (GraphPad software, La Jolla, CA). *P* values ≤ 0.05 were considered statistically significant.

## SUPPLEMENTARY MATERIALS FIGURES AND TABLES


