# Supplementary material for: The tissue inhibitor of metalloproteinases-1 (TIMP-1) promotes survival and migration of acute myeloid leukemia cells through CD63/PI3K/Akt/p21 signaling
Source: Oncotarget. 2016 Nov 26;8(2):2261–74. doi: 10.18632/oncotarget.13664 (PMC5356797; doi:10.18632/oncotarget.13664)
Supplement: Supplementary file 1 [file oncotarget-08-2261-s001.pdf]

The tissue inhibitor of metalloproteinases-1 (TIMP-1) promotes survival and migration of acute myeloid leukemia cells through CD63/PI3K/Akt/p21 signaling

Supplementary Materials

Table 1: Patient characteristics

# PT	AGE (years)	SEX	CELL SOURCE	SUBCLASS (FAB)	Karyotype	Molecular genetics	WBC (cells/µL)
1	66	M	PB	M0/M1	Normal	wt	148,900
2	33	M	PB	NOS	t(6;9)(p23;q34)	wt	20,560
3	73	M	BM	M2	Normal	wt	1,580
4	60	F	BM	M4	t(4)	NPM1^{+}, FLT3^{+}	129,000
5	77	F	PB	M3	t(15,17)	PML/RARα	99,300
6	90	M	PB	NOS	NA	wt	129,000
7	19	M	PB	M0	t(6;11)	wt	100,000
8	57	F	PB	NOS	Normal	FLT3-ITD^{+}, DNMT3A^{+}, WT1	78,500
9	70	M	PB	M2	NA	FLT3-ITD^{+}, NPM1^{+}	64,250
10	42	F	PB	M4	Normal	FLT3-ITD^{+}	20,300
11	67	M	BM	M0-M1	+(8)	wt	1,200
12	69	M	PB	NOS	Normal	FLT3-ITD^{+}	88,680
13	72	M	BM	NOS	Normal	WT1	260,000
14	66	F	BM	M0/M1	Normal	WT1	141,700
15	47	F	BM	NOS	Normal	FLT3-ITD^{+}	89,380
16	42	M	BM	M2	+(8)	wt	7,436
17	26	M	BM	M2	trisomy 22	DNMT3A	40,000
18	72	F	BM	NOS	NA	WT1	13,800

Abbreviations: FAB, French-American-British classification of acute myeloid leukemia; WBC White blood cells; PB, peripheral blood; BM, bone marrow; NPM, nucleophosmin; FLT3 ITD, fms-like tyrosine kinase 3 internal tandem duplication; DNMT3A DNA-methyltransferase 3A; WT1 Wilms Tumor 1; NOS Not Otherwise Specified; NA Not Available; wt wild type.

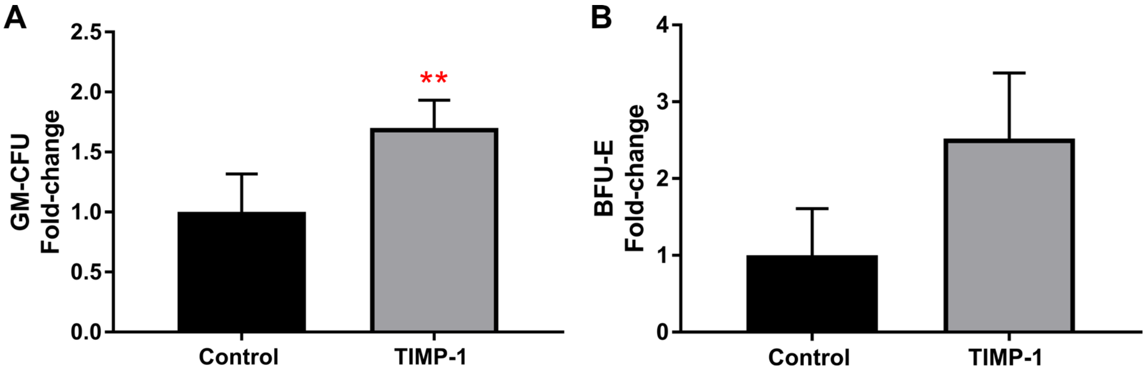

Supplementary Figure S1: Effects of TIMP-1 on CFU-GM/BFU-E growth from AML-derived cells. Circulating leukemic blasts were isolated from AML patients and cultured in semisolid medium in the presence of TIMP-1. After 14 days, the total CFU-L output was assessed as above described. (**A**) The CFU-GM growth of the AML-derived leukemic cells was significantly stimulated by TIMP-1 (100 ng/ml, $^{**}p \leq 0.01$) (n = 12). The mean number of CFU-GM colonies in untreated (0 ng/ml) and treated (100 ng/ml) AML samples was 17.5 ± 5.5 vs 28 ± 9.6, respectively. (**B**) The growth of BFU-E showed the same pattern displayed by CFU-GM but not significantly (n = 5). The mean number of BFU-E colonies in untreated (0 ng/ml) and treated (100 ng/ml) AML samples was 7.4 ± 4.49 vs 11 ± 4.7, respectively. The results are expressed as growth fold change versus untreated control samples.

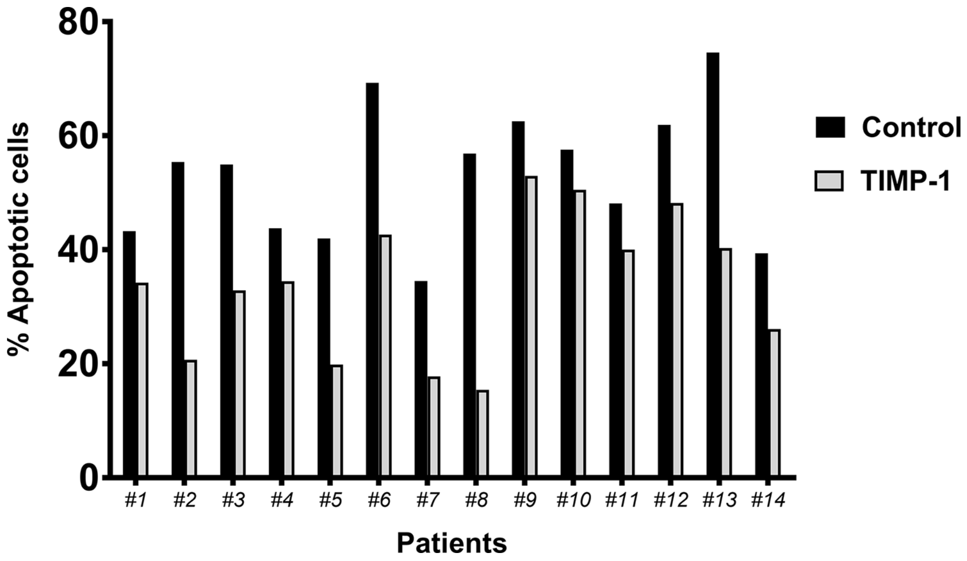

Supplementary Figure S2: Effects of TIMP-1 on survival in 14 AML patients. AML cells from 14 patients were in vitro treated for 2 days with TIMP-1 and the percentage of cell viability was assessed after AnnexinV/PI staining, as described in methods. For each patient the percentage of apoptotic cells was reported in the absence (black columns) or in presence of TIMP-1 (grey columns).

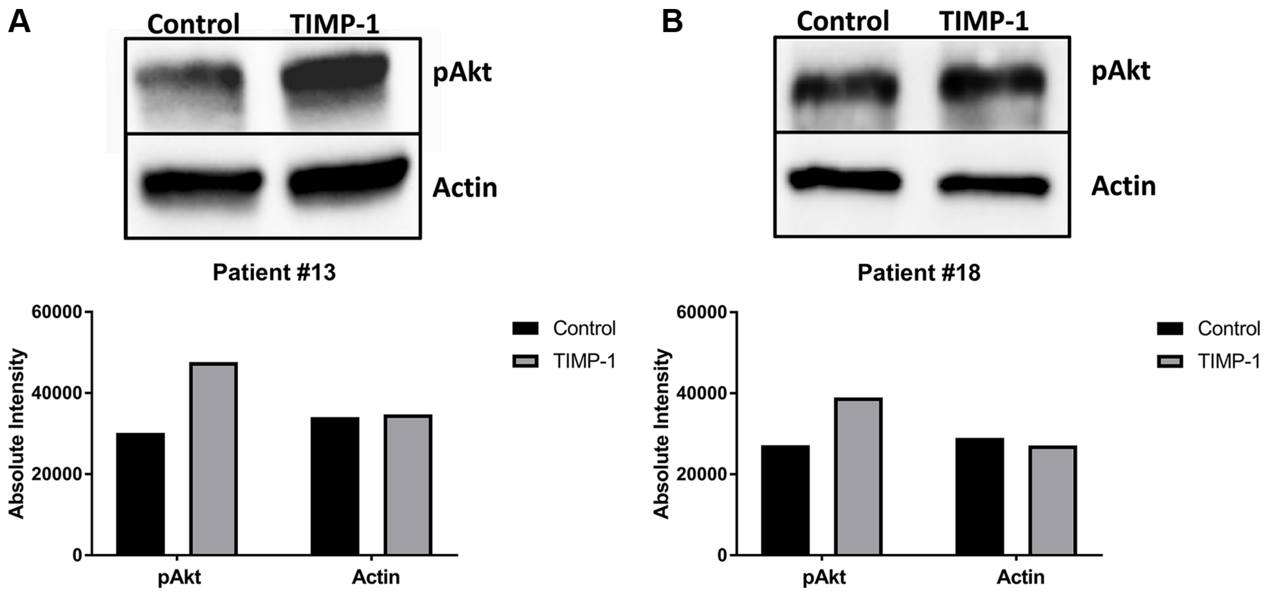

Supplementary Figure S3: Western blot analysis and absolute quantification of pAkt in AML cells after TIMP-1 treatment. Representative Western-blot bands of leukemic blasts from two AML patients. Western-blot bands for pAkt (first lane) in AML cells untreated (left band, control) and TIMP-1 treated (right band, TIMP-1) for patient #13 (**A**) and patient #18 (**B**). Graphic representation of quantification for pAkt expression and the relative β-actin (data shown below).

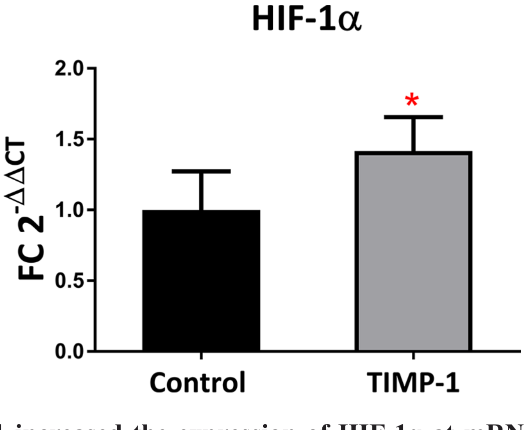

Supplementary Figure S4: TIMP-1 increased the expression of HIF-1α at mRNA level. After 24 hours, total RNA was extracted and the correspondent levels HIF-1α was assessed by Real-Time PCR. Results was expressed as fold-change taking the value of untreated cells as 1 (*$p \leq 0.05$; $n = 5$).
